# Measurement properties of utility-based health-related quality of life measures in cardiac rehabilitation and secondary prevention programs: a systematic review

**DOI:** 10.1007/s11136-024-03657-5

**Published:** 2024-07-03

**Authors:** Norma B. Bulamu, Lemlem G. Gebremichael, Sonia Hines, Christine Mpundu-Kaambwa, Vincent Pearson, Hila A. Dafny, Maria Alejandra Pinero de Plaza, Alline Beleigoli, Billingsley Kaambwa, Jeroen M. Hendriks, Robyn A. Clark

**Affiliations:** 1 Flinders Health and Medical Research Institute, College of Medicine and Public Health, Flinders University, Adelaide, Australia; 2 Caring Futures Institute, College of Nursing and Health Sciences, Flinders University, University Drive, South Australia (SA), Bedford Park, Adelaide, 5042 Australia; 3 Mparntwe Centre for Evidence in Health, Flinders University: A JBI Centre of Excellence, Alice Springs, Australia; 4 Flinders Rural and Remote Health, College of Medicine and Public Health, Flinders University, Alice Springs, Australia; 5 Health and Social Care Economics Group, Caring Futures Institute, College of Nursing and Health Sciences, Flinders University, Adelaide, Australia; 6 Economics of Global Health & Infectious Disease Unit, Melbourne Health Economics, Centre for Health Policy, School of Population and Global Health, The University of Melbourne, Melbourne, Australia; 7 JBI, School of Public Health, The University of Adelaide, Adelaide, Australia; 8 Centre of Research Excellence: Frailty and Healthy Ageing, The University of Adelaide, Adelaide, South Australia 5000 Australia; 9 Centre for Heart Rhythm Disorders, University of Adelaide and Royal Adelaide Hospital, Adelaide, Australia; 10 Southern Adelaide Local Health Network, Bedford Park, South Australia (SA) 5042 Australia

**Keywords:** Cardiac rehabilitation and secondary prevention programs, International classification of functioning disability and health, Preference-based measures, Measurement properties, Patient-reported outcome measures

## Abstract

**Purpose:**

To identify utility-based patient-reported outcome measures (PROMs) for assessing health-related quality of life (HRQoL) in cardiac rehabilitation and secondary prevention programs (CR) and appraise existing evidence on their measurement properties. Secondly, to link their items to the International Classification of Functioning Disability and Health (ICF) and the International Consortium of Health Outcome Measures (ICHOM) domains for cardiovascular disease (CVD).

**Methods:**

Eight databases were searched. The review followed the COSMIN and JBI guidelines for measurement properties systematic reviews and PRISMA 2020 reporting guidelines. Non-experimental and observational empirical studies of patients ≥ 18 years of age with CVD undergoing CR and assessed quality of life (QoL) or HRQoL using utility-based PROMs or one accompanied by health state utilities were included.

**Results:**

Nine PROMs were identified with evidence on measurement properties for three measures: the German translations of SF-12, EQ-5D-5L, and MacNew heart disease HRQoL questionnaire. There was moderate quality evidence for responsiveness and hypothesis testing of the SF-12 and EQ-5D-5L, and high-quality evidence for responsiveness and hypothesis testing for the MacNew.

All items of SF-12 and EQ-5D were linked to ICF categories, but four items of the MacNew were not classified or defined. All the PROM domains were mapped onto similar constructs from the ICHOM global sets.

**Conclusion:**

Three utility-based PROMs validated in CR were identified: the German versions of the EQ-5D and SF-12 and the MacNew questionnaire. These PROMs are linked to a breadth of ICF categories and all ICHOM global sets. Additional validation studies of PROMs in CR are required.

**Supplementary Information:**

The online version contains supplementary material available at 10.1007/s11136-024-03657-5.

## Introduction

The World Health Organization (WHO) defines cardiac rehabilitation and secondary prevention programs as ‘the sum of activity and interventions required to ensure the best possible physical, mental, and social conditions so that patients with chronic or post-acute cardiovascular disease may, by their efforts, preserve or resume their proper place in society and lead an active life’ [1]. Cardiac rehabilitation and secondary prevention programs (CR) are recommended for patients diagnosed with coronary heart disease, heart failure, heart valve disease and following cardiac surgery, including coronary artery bypass graft and following a cardiac event [2]. Cardiac rehabilitation and secondary prevention programs aim to delay disease progression or prevent future cardiac events, also referred to as secondary prevention. Secondary prevention includes lifestyle interventions for risk factor management, such as healthy eating, exercise, weight management, and psychosocial support, including monitoring of patient-reported outcomes [3].

Patient-reported outcomes encompass any report on a patient’s condition as reported by the patient [4]. The assessment of patient-reported outcomes is increasingly important as part of routine patient monitoring and as a quality indicator for treatment programs such as CR [3, 5]. In addition, patient-reported outcomes are a key outcome measure in economic evaluation studies assessing the cost-effectiveness of different healthcare interventions. A recent international study on the cost of CR reported average cost per patient ranging from US$731.54 in the United Kingdom to US$1023.99 in Australia and US$5016.60 in the United States of America [6]. Reported healthcare expenditure on cardiovascular diseases is significant, amounting to AU$12.7 billion in Australia [7] and, £7.4 billion in the United Kingdom [8] in 2019/20 and €155 billion in the European Union in 2021 [9]. With increasing CVD prevalence and morbidity globally [10], rising expenditure is certain, and therefore, the efficient allocation of these resources must be considered. The use of PROMs has been on the rise, and there is a growing demand for cost-utility analysis to evaluate the cost-effectiveness of healthcare programs. This trend aligns with recommendations from influential decision-making bodies, including the Pharmaceutical Benefits Advisory Committee (PBAC) and the Medical Services Advisory Committee (MSAC) in Australia, as well as the National Institute for Health and Care Excellence (NICE) in the UK [11, 12]. There are different types of economic evaluations depending on how the outcomes are assessed, and health-related quality of life (HRQoL), when assessed using utility-based patient-reported outcome measures (PROMs), also known as preference-based PROMs, is applied in cost-utility analysis [13, 14]. Preference-based or utility-based PROMs are comprised of an HRQoL assessment accompanied by a utility algorithm, which is an indication of the preferences of the different health states generated by completing the assessment. By applying the utility weights, the scores obtained from such PROMs, referred to as utility scores, are used to generate quality adjusted life years (QALYs), the outcome measure in cost-utility analysis [13, 14]. The QALY is a composite measure of the quantity of life accrued by a given intervention (usually calculated using survival analysis) and the utility obtained from that life (utility scores obtained when a utility-based PROM assesses HRQoL). Examples of such PROMs include generic measures such as the Euroqol 5-dimensions measures, EQ-5D-3L and EQ-5D-5L [15], the Short-Form 6-Dimensions (SF-6D) [16] and disease-specific measures such as the MacNew heart disease HRQoL questionnaire [17].

Cardiac rehabilitation and secondary prevention programs are an evidence-based intervention that improves the HRQoL of people with CVD; therefore, HRQoL is a recommended measured outcome in this population. Although several PROMs have been validated in populations with cardiovascular disease and CR programs, there is a limited understanding of the suitability of these PROMs for use in cost-utility analysis studies. It is, therefore, important to identify the most suitable utility-based PROM in this population by assessing the quality of its measurement properties and its relevance to the needs of that specific population. This will facilitate accurate assessment of the cost-utility of CR programs and inform decision making.

With the increasing number of PROMs, guidance on the choice of PROM to be used in a specific population is required by mapping their content to internationally recommended patient-reported outcomes to be assessed in each population. The International Classification of Functioning, Disability and Health (ICF) is a recognized tool for comparing different PROMs [18, 19]. It is a bio-psychosocial framework of health developed by the World Health Organization for measuring health and disability at both individual and population levels across different categories: Body functions, Body structures, Activities and participation, Environmental factors, and Personal factors [20]. In addition, to achieve value-based care, the International Consortium for Health Outcomes Measurement (ICHOM) has defined key patient-reported outcomes that are important to and should be monitored in patients affected by different diseases, including CVD [21].

Therefore, this review aimed to identify utility-based PROMs that have been validated for use in a population undergoingCR. To assess their suitability for this population, the PROMs were mapped onto the ICF and the PRO global sets for cardiovascular disease, including atrial fibrillation [22], heart failure [23], heart valve disease and coronary artery disease [24] developed by ICHOM.

## Review question(s)

1. Which utility-based PROMs have been validated for assessing HRQoL in patients attending cardiac rehabilitation and secondary prevention programs?
2. How does the content of these measures compare to the ICF framework, and do they address the domains recommended by ICHOM for individuals with CVD?

## Methods

This review was registered with PROSPERO (CRD42022349395) and conducted following the JBI methodology for systematic reviews of measurement properties [25]. The full protocol for the conduct of this review has been published in detail elsewhere [26], and a summary is presented below.

### Inclusion criteria

This review considered studies in adults ≥ 18 years of age eligible for a cardiac rehabilitation and secondary prevention program, assessing quality of life or HRQoL using a generic, disease-specific, or population-specific utility-based health-related PROM or PROMs accompanied by a scoring algorithm to generate utility scores. Studies were considered for inclusion if they assessed one or more aspects related to the measurement properties, development (to assess content validity), or interpretability of the PROM. Included studies reported on at least one of the following properties: 1) reliability, encompassing internal consistency, reliability, and measurement error, 2) validity, including structural validity, content validity, and construct validity, and 3) responsiveness. The COSMIN definitions for these measurement properties and the tests to assess them are provided in Supplementary Table S1.

### Types of studies

Studies of quasi-experimental designs, before and after studies, analytical observational studies, including prospective and retrospective cohort studies, case–control studies, and cross-sectional studies were considered.

### Search strategy

We employed a three-step search approach, commencing with an initial exploration of MEDLINE (via Ovid) and CINAHL (via EBSCO) to pinpoint relevant articles pertaining to the subject. Subsequently, we extracted text words and index terms from pertinent articles to formulate a comprehensive search strategy for use across other databases. We also examined the reference lists of included studies to identify any relevant supplementary studies. A search strategy was developed based on COSMIN-recommended search filters and previously published research in patients undergoing a cardiac rehabilitation and secondary prevention program [27] and assessing HRQoL [28]. This search strategy is provided in supplementary data, Table S2. Studies published from database inception to 30th Sept 2022 were included.

#### Instrument

For the ‘type of instrument’ concept, search filters developed by the Patient-Reported Outcomes Measurement Group (PROM Group) at the University of Oxford were used to find studies that evaluated PROMs [29].

#### Measurement properties

The highly sensitive validated search filters developed by the COSMIN initiative in PubMed were used to find measurement property studies. Translation of the original PubMed filter to Ovid MEDLINE by Macquarie University was employed [29].

#### Databases

The Databases searched were MEDLINE (Ovid), Emcare (Ovid), Embase (Ovid), Scopus (Elsevier), CINAHL (EBSCO), Web of Science Core Collection (Clarivate), lnformit, PsyclNFO (Ovid) and REHABDATA. Unpublished studies/grey literature was searched in Dissertations and Theses Global, WorldCat, Health, Psychosocial Instruments (HaPI) database, and a list of information sources specific to PROMS collated by the PROMS Group at the University of Oxford (e.g. organizations and research groups; journals; royal colleges and relevant links).

### Study selection

Two independent reviewers screened the studies (abstracts and titles and then full texts) in Covidence (NB, LG, CMK, HD, VP, MAPP), and conflicts were resolved by involving a third reviewer (AB, SH, JH and RC). Search results and the study inclusion process were presented in a Preferred Reporting Items for Systematic Reviews and Meta-analyses (PRISMA) flow diagram [30].

### Assessment of methodological quality of the study

The quality of each study was appraised against the COSMIN Risk of Bias checklist [31]. Two independent reviewers completed the checklist for methodological quality, and a third reviewer was involved in any disagreements. Studies were rated as ‘very good’, ‘adequate’, ‘doubtful’ or ‘inadequate’ quality. An overall rating was assigned based on the lowest rating for any standards assessed in the checklist [32]. Data extraction and synthesis were conducted regardless of methodological quality, with the impact of including studies with ‘doubtful’ or ‘inadequate’ ratings assessed in the sensitivity analysis. However, studies with ‘inadequate’ evidence on content validity were excluded from further assessment in the review at this stage [31].

### Data extraction

Two independent reviewers (NB, LG, SH, HD, VP) extracted the data using modified overview tables and templates from appendices 3–6 of the COSMIN manual [33], and any disagreements were resolved by involving a third reviewer.

### Data synthesis

Data on measurement properties for each PROM was synthesised and evaluated by two independent reviewers (NBB, HD, SH) and conflicts were resolved by a third reviewer (CMK and BK). The quality of each measurement property reported in the included studies was qualitatively summarised, and a narrative synthesis was provided.

The aggregated results were compared against the criteria for good measurement properties to determine whether the measurement property of the PROM was sufficient (+), insufficient (–), inconsistent ( ±), or indeterminate (?) [33, 34]. A positive rating was assigned if the authors provided sufficient evidence that a particular property has been satisfied, negative if not and indeterminate if no information was provided.

The quality of the evidence generated for each measurement property was also graded as high, moderate, low, or very low using the Grading of Recommendations Assessment, Development, and Evaluation (GRADE) approach [31, 33].

### Mapping PROM items to ICF categories and domains to ICHOM global sets

To evaluate the content validity of each PROM and its relevance to the needs of patients undergoing cardiac rehabilitation and secondary prevention programs, the content/items of the PROMs were mapped onto the ICF using standardized linking rules and their domains were compared to the domains recommended by the ICHOM for cardiovascular disease.

## Results

Supplementary Figure S1 illustrates the screening and full-text review to identify the relevant studies for inclusion. 

### Study characteristics

This review found ten eligible studies conducted between 2004 and 2019, with the majority (4) undertaken in Germany. All studies were observational except for one randomised control trial [35] (see Table S3). Nine utility-based PROMs were identified; five language translations of the MacNew heart disease HRQoL questionnaire (MacNew): French [36], Portuguese [37], Italian [38], Persian [35] and German [39, 40], two versions of the 12-Item Short Form Health Survey (SF-12), English [41] and German [42] and the German translation of EQ-5D-3L [43] and EQ-5D-5L [44]. The SF-36 was the predominant PROM (7 studies) against which these PROMs were compared for convergent validity.

The EQ-5D is a utility-based health status measure with general population value sets from several countries, including Australia [45–47]. Although both the SF-12 and MacNew questionnaires are not stand-alone preference-based measures like EQ-5D, utility scores can be obtained from responses to SF-12 using its utility system, the SF-6D [48] and from MacNew using the health state classification system developed by Kularatna et al. [17]. As such, these two PROMs were included in this review.

### Assessment of methodological quality

Methodological quality was assessed for each study against the COSMIN Risk of Bias checklist [31]. These results are presented in Table 1. Nine studies assessed hypothesis testing for construct validity [35–40, 42–44], seven studies assessed internal consistency [35–40, 44], and responsiveness [35, 37, 39–43], six assessed structural validity [36–40, 44] while four studies assessed reliability [35–37, 43] and only one study assessed criterion validity [43]. None of the studies assessed any of the following properties, PROM development, content validity, cross‐cultural validity, measurement invariance or measurement error.

**Table 1:** Quality of studies on measurement properties—COSMIN checklist

PROM	Structural validity	Internal consistency	Reliability	Criterion validity	Construct validity		Responsiveness			
					Convergent validity	Known groups validity	Comparison with gold standard	Comparison with other instruments	Comparison between subgroups	Comparison pre and post intervention
English SF-12 [41] Agreement = 1.0	NA	NA	NA	NA	NA	NA	NA	NA	NA	Very good
French SF-12 [42] Agreement = 1.0	NA	NA	NA	NA	Very good	NA	Very good	Very good	NA	Very good
German EQ-5D-3L [43] Agreement = 1.0	NA	NA	Very good	Very good	Very good	Adequate	NA	adequate	Adequate	Adequate
German EQ-5D-5L [44] Agreement = 1.0	Very good	Very good	NA	NA	Very good	Very good	NA	NA	NA	NA
Portuguese MacNew [37] Agreement = 1.0	Inadequate	Very good	Adequate	NA	Very good	Very good	NA	NA	NA	Doubtful
French MacNew [36] Agreement = 0.86	Adequate	Very good	Adequate	NA	Very good	Vvery good	NA	NA	NA	NA
Italian MacNew [38] Agreement = 1.0	Adequate	Very good	NA	NA	Very good	Very good	NA	NA	NA	NA
Persian MacNew [35] Agreement = 0.73	NA	Inadequate	Very good	NA	Very good	Adequate	NA	NA	NA	Very good
German MacNew [40] Agreement = 0.85	Inadequate	Very good	NA	NA	Very good	Very good	NA	NA	NA	Very good
German MacNew [39] Agreement = 0.84	Very good	Very good	NA	NA	Adequate	NA	NA	NA	NA	Doubtful

*NA* = Not assessed, Agreement = Cohen’s Kappa

Hypothesis testing for construct validity was *very good* except in three studies where it was *adequate* because there was no evidence that the comparator instrument for assessing convergent validity had been validated in the study population [39] and because the statistical method for assessing known groups was not stated but assumed by the reviewers to be appropriate [35, 43]. The internal consistency was *very good* in six of the seven studies assessing these properties and inadequate in one study [35] where no information was provided about whether other specific internal consistency statistics or IRT-based scores such as standard error were calculated. Responsiveness was *very good* in four of the seven studies [35, 40–42], *adequate* in one study [43] because the statistical methods were not stated and *doubtful* in two studies where the intervention was not adequately described [37, 39]. Factor analysis was performed on each sub-scale separately for studies assessing structural validity. Structural validity was *very good* in two studies that applied confirmatory factor analysis [39, 44], *adequate* in the two studies that applied exploratory factor analysis [36, 38] and *inadequate* in two studies where factor analysis was not used [37] and where the sample size was below 5 × the number of items tested [37, 40]. The assessment of reliability was *very good* in two of the four studies [35, 43] and *adequate* in two studies because the intra-class correlation was calculated but the model was not described [36, 37].

### Data synthesis

Although six studies applied the MacNew, it was administered in five different language translations, and these studies could not be pooled together in a meta-analysis. A narrative synthesis based on the GRADE assessment is therefore provided with details in Table 2. Responsiveness of the English version of the SF-12 (*n* = 65) was rated as sufficient (+); however, the quality of evidence was low because only one study was identified [41]. Conversely, for the French version of SF-12, the quality of evidence was moderate for responsiveness and hypothesis testing; although only one study because the sample size was large (*n* = 2441) [42]. Rating for reliability, criterion validity, hypothesis testing and responsiveness of the German version of EQ-5D-3L was sufficient (+), but the quality of evidence was low as only one study (*n* = 114) was identified [43]. On the other hand, the quality of evidence for structural validity, internal consistency, hypothesis testing and responsiveness of the German version of EQ-5D-5L was moderate because of this study’s significantly larger sample size (*n* = 3225) [44]. For the MacNew, assessment of structural validity, internal consistency and hypothesis testing was rated as sufficient (+); however, the quality of evidence was low for the Portuguese (*n* = 200) [37], French (*n* = 323) [36], Italian (*n* = 298) [38] and Persian (*n* = 60) [35] translations. The quality of evidence was high for the German version as two studies [39, 40] with significantly high sample size (overall *n *= 5781) were included. Reliability was rated as sufficient in all except the Italian version [38], where it was not assessed. The quality of this evidence was low except for the German version [39, 40], where it was moderate. Responsiveness was sufficient in the German and Persian versions [35], but the quality of evidence was low for the Persian version because only one study was included.

**Table 2:** GRADE—Quality of the evidence for measurement properties of the PROMS

		Structural validity	Internal consistency	Reliability	Criterion validity	Hypothesis testing for construct validity	Responsiveness
English SF-12 [41]	Overall rating	NA	NA	NA	NA	NA	+
	Quality of evidence	NA	NA	NA	NA	NA	Low
French SF-12 [42]	Overall rating	NA	NA	NA	NA	+	+
	Quality of evidence	NA	NA	NA	NA	moderate	Moderate
German EQ-5D-3L [43]	Overall rating	NA	NA	+	+	+	+
	Quality of evidence	NA	NA	Low	Low	Low	Low
German EQ-5D-5L [44]	Overall rating	+	+	NA	NA	+	NA
	Quality of evidence	Moderate	moderate	NA	NA	moderate	NA
Portuguese MacNew [37]	Overall rating	-	+	+	NA	+	+
	Quality of evidence	Very low	Low	Low	NA	Low	Low
French MacNew [36]	Overall rating	+	+	+	NA	+	NA
	Quality of evidence	Low	moderate	Low	NA	moderate	NA
Italian MacNew [38]	Overall rating	+	+	NA	NA	+	NA
	Quality of evidence	Low	Low	NA	NA	Low	NA
Persian MacNew [35]	Overall rating	NA	+	+	NA	+	+
	Quality of evidence	NA	Low	Low	NA	Low	Low
German MacNew [39, 40]	Overall rating	+	+	NA	NA	+	+
	Quality of evidence	Moderate	High	NA	NA	High	High

*NA* = Not assessed; Agreement between assessors was 1.0 for the overall rating and the quality of the evidence

### Mapping PROM items to ICF categories and domains to ICHOM global sets

Nine different PROMs were identified with four core questionnaires: MacNew, EQ-5D-5L, EQ-5D-3L and the SF-12. Because both EQ-5D-3L and EQ-5D-5L only differ in the levels and not the items, this was treated as one measure for the linking and mapping exercise. Linking followed the linking rules for PROMs developed by Cieza et al., 2005 [49].Cieza et al. published the ICF linking results for the SF-12 and therefore, this review reproduces linking results from that original paper (Table S4) [49]. In this review, we linked the MacNew and EQ-5D, reported in Tables 4 and 5.

**Table 3:** Matching to ICHOM global sets

Item	ICHOM global sets
MacNew	
1. In general, how much of the time during the last 2 weeks have you felt frustrated, impatient, or angry?	Coronary artery disease—HRQoL Heart valve disease—Quality of life, Mental health Heart failure—Psychosocial health Atrial fibrillation—HRQoL, Functioning (Physical, Emotional and Cognitive)
2. How often during the last 2 weeks have you felt worthless or inadequate?	Coronary artery disease—HRQoL, depression Heart valve disease—Quality of life, Mental health Heart failure—Psychosocial health Atrial fibrillation—HRQoL, Functioning (Physical, Emotional and Cognitive)
3. In the last 2 weeks, how much of the time did you feel very confident and sure that you could deal with your heart problem?	Coronary artery disease—HRQoL Heart valve disease—Quality of life, Mental health Heart failure—Psychosocial health Atrial fibrillation—HRQoL, Functioning (Physical, Emotional and Cognitive)
4. In general how much of the time did you feel discouraged or down in the dumps during the last 2 weeks?	Coronary artery disease—HRQoL, depression Heart valve disease—Quality of life, Mental health Heart failure—Psychosocial health Atrial fibrillation—HRQoL, Functioning (Physical, Emotional and Cognitive)
5. How much of the time during the past 2 weeks did you feel relaxed and free of tension?	Coronary artery disease—HRQoL Heart valve disease—Quality of life, Mental health Heart failure—Psychosocial health Atrial fibrillation —HRQoL, Functioning (Physical, Emotional and Cognitive)
6. How often during the last 2 weeks have you felt worn out or low in energy?	Coronary artery disease—HRQoL, functional status Heart valve disease—Quality of life, Impact on mental health and daily activities Heart failure—Psychosocial health Atrial fibrillation—HRQoL, Functioning (Physical, Emotional and Cognitive)
7. How happy, satisfied, or pleased have you been with your personal life during the last 2 weeks?	Coronary artery disease—HRQoL Heart valve disease—Quality of life, Mental health Heart failure—Psychosocial health Atrial fibrillation—HRQoL, Functioning (Physical, Emotional and Cognitive)
8. In general, how often during the last 2 weeks have you felt restless, or as if you were having difficulty trying to calm down?	Coronary artery disease—HRQoL Heart valve disease—Quality of life, Mental health Heart failure—Psychosocial health Atrial fibrillation—HRQoL, Functioning (Physical, Emotional and Cognitive)
9. How much shortness of breath have you experienced during the last 2 weeks while doing your day-to-day physical activities?	Coronary artery disease—HRQoL, dyspnea Heart valve disease—Quality of life Heart failure—Symptom control, Vital status Atrial fibrillation—HRQoL, Symptom severity
10. How often during the last 2 weeks have you felt tearful or like crying?	Coronary artery disease—HRQoL, depression Heart valve disease—Quality of life, Mental health Heart failure—Psychosocial health Atrial fibrillation—HRQoL, Functioning (Physical, Emotional and Cognitive)
11. How often during the last 2 weeks have you felt as if you are more dependent than you were before your heart problem?	Coronary artery disease—HRQoL, functional status Heart valve disease—Quality of life, Impact on mental health and daily activities Heart failure—Independence Atrial fibrillation—HRQoL, Functioning (Physical, Emotional and Cognitive)
12. How often during the last 2 weeks have you felt that you were unable to do your usual social activities or social activities with your family?	Coronary artery disease—HRQoL, functional status Heart valve disease—Quality of life, Impact on mental health and daily activities Heart failure—Psychosocial health, Activities of daily living Atrial fibrillation—HRQoL, Functioning (Physical, Emotional and Cognitive), Ability to work
13. How often during the last 2 weeks have you felt as if others no longer have the same confidence in you as they did before your heart problem?	Coronary artery disease—HRQoL Heart valve disease—Quality of life, Mental health Heart failure—Psychosocial health Atrial fibrillation—HRQoL, Functioning (Physical, Emotional and Cognitive)
14. How often during the last 2 weeks have you experienced chest pain while doing your day-to-day activities?	Coronary artery disease—HRQoL, angina Heart valve disease—Quality of life Heart failure—Symptom control Atrial fibrillation—HRQoL, Symptom severity
15. How often during the last 2 weeks have you felt unsure of yourself or lacking in self-confidence?	Coronary artery disease—HRQoL Heart valve disease—Quality of life, Mental health Heart failure—Psychosocial health Atrial fibrillation—HRQoL, Functioning (Physical, Emotional and Cognitive)
16. How often during the last 2 weeks have you been bothered by aching or tired legs?	Coronary artery disease—HRQoL Heart valve disease—Quality of life Heart failure—Psychosocial health, Activities of daily living Atrial fibrillation—HRQoL, Functioning (Physical, Emotional and Cognitive)
17. During the last 2 weeks, how much have you been limited in doing sports or exercise as a result of your heart problem?	Coronary artery disease—HRQoL, functional status Heart valve disease—Quality of life, Impact on mental health and daily activities Heart failure—Psychosocial health, Activities of daily living Atrial fibrillation—HRQoL, Functioning (Physical, Emotional and Cognitive), Exercise tolerance
18. How often during the last 2 weeks have you felt apprehensive or frightened?	Coronary artery disease—HRQoL Heart valve disease—Quality of life, Mental health Heart failure—Psychosocial health Atrial fibrillation—HRQoL, Functioning (Physical, Emotional and Cognitive)
19. How often during the last 2 weeks have you felt dizzy or lightheaded?	Coronary artery disease—HRQoL Heart valve disease—Quality of life Heart failure—Symptom control Atrial fibrillation—Symptom severity
20. In general, during the last 2 weeks how much have you been restricted or limited as a result of your heart problem?	Coronary artery disease—HRQoL, functional status Heart valve disease—Quality of life, Impact on mental health and daily activities Heart failure—Independence, Activities of daily living Atrial fibrillation—HRQoL, Functioning (Physical, Emotional and Cognitive)
21. How often during the last 2 weeks have you felt unsure as to how much exercise or physical activity you should be doing?	Coronary artery disease—HRQoL, functional status Heart valve disease—Quality of life, Impact on mental health and daily activities Heart failure –Activities of daily living Atrial fibrillation—Functioning (Physical, Emotional and Cognitive), Exercise tolerance
22. How often during the last 2 weeks have you felt as if your family is being over-protective toward you?	Coronary artery disease—HRQoL Heart valve disease—Quality of life, Mental health Heart failure—Psychosocial health, Independence Atrial fibrillation—Functioning (Physical, Emotional and Cognitive)
23. How often during the past 2 weeks have you felt as if you were a burden to others?	Coronary artery disease—HRQoL Heart valve disease—Quality of life, Mental health Heart failure—Psychosocial health, Independence Atrial fibrillation—Functioning (Physical, Emotional and Cognitive)
24. How often during the past 2 weeks have you felt excluded from doing things with other people because of your heart problem?	Coronary artery disease—HRQoL Heart valve disease—Quality of life, Impact on mental health and daily activities Heart failure—Psychosocial health, Independence Atrial fibrillation—HRQoL, Functioning (Physical, Emotional and Cognitive)
25. How often during the past 2 weeks have you felt unable to socialize because of your heart problem?	Coronary artery disease—HRQoL Heart valve disease—Quality of life, Impact on mental health and daily activities Heart failure—Psychosocial health, Independence, Activities of daily living Atrial fibrillation—HRQoL, Functioning (Physical, Emotional and Cognitive)
26. In general, during the last 2 weeks how much have you been physically restricted or limited as a result of your heart problem?	Coronary artery disease—HRQoL, Functional status Heart valve disease—Quality of life, Impact on mental health and daily activities Heart failure—Independence, Activities of daily living Atrial fibrillation—HRQoL, Functioning (Physical, Emotional and Cognitive)
27. How often during the last 2 weeks have you felt your heart problem limited or interfered with sexual intercourse?	Coronary artery disease—HRQoL Heart valve disease—Quality of life, Impact on mental health and daily activities Heart failure—Independence, Activities of daily living Atrial fibrillation—HRQoL, Functioning (Physical, Emotional and Cognitive)
EQ-5D	
Mobility I have no problems in walking about I have slight problems I have moderate problems I have severe problems I am unable to walk about	Coronary artery disease—Functional status Heart valve disease—Impact on mental health and daily activities Heart failure –Activities of daily living Atrial fibrillation—Functioning (Physical, Emotional and Cognitive),
Self-care I have no problems washing or dressing myself I have slight problems I have moderate problems I have severe problems I am unable to wash or dress myself	Coronary artery disease—Functional status Heart valve disease—Impact on mental health and daily activities Heart failure—Psychosocial health, Activities of daily living, Independence Atrial fibrillation—HRQoL,
Usual activities (e.g. work, study, housework, family or leisure activities) I have no problems doing my usual activities I have slight problems doing I have moderate problems doing I have severe problems doing I am unable to do my usual activities	Coronary artery disease—Functional status Heart valve disease—Impact on mental health and daily activities Heart failure—Psychosocial health, Activities of daily living Atrial fibrillation—HRQoL, Functioning (Physical, Emotional and Cognitive), Independence
Pain/discomfort I have no pain or discomfort I have slight pain or discomfort I have moderate pain or discomfort I have severe pain or discomfort I have extreme pain or discomfort	Coronary artery disease—HRQoL Heart valve disease—Quality of life Heart failure—Activities of daily living Atrial fibrillation—HRQoL
Anxiety/depression I am not anxious or depressed I am slightly anxious or depressed I am moderately anxious I am severely anxious or depressed I am extremely anxious	Coronary artery disease—HRQoL Heart valve disease—Mental health Heart failure—Psychosocial health Atrial fibrillation —Functioning (Physical, Emotional and Cognitive)

**Table 4:** Linking MacNew questionnaire to the International Classification of Functioning, Disability and Health (ICF)

Rater	ICF category	Level 1	Level 2	Level 3	Level 4/additional info
1. In general, how much of the time during the last 2 weeks have you felt frustrated, impatient, or angry?					
	Body functions—Mental functions	Specific mental functions	b152 Emotional functions	b1522 Range of emotion	Mental functions that produce the spectrum of experience of arousal of affect or feelings such as love, hate, anxiousness, sorrow, joy, fear and anger)
2. How often during the last 2 weeks have you felt worthless or inadequate?					
Rater 1&2	Body function—Mental function	Specific mental functions	b180 experience of self and time functions	b1800 experience of self	Specific mental functions of being aware of one's own identity and one's position in the reality of one's environment around oneself
3. In the last 2 weeks, how much of the time did you feel very confident and sure that you could deal with your heart problem?					
Rater 1	Not classified—NC				
Rater 2	Body function—Mental function	Global mental function	b126 Temperament and personality functions	b1266 Confidence	
4. In general how much of the time did you feel discouraged or down in the dumps during the last 2 weeks?					
Rater 1	Body function—Mental functions	Global mental function	b126 Temperament and personality functions	b1269 Temperament and personality functions, unspecified	Temperament and personality functions, unspecified Temperament and personality functions functions of emotional stability
Rater 2	Body function—Mental functions	Global mental function	b126 Temperament and personality functions	b1268 Other specified temperament and personality functions	Discouraged or down in the dump
5. How much of the time during the past 2 weeks did you feel relaxed and free of tension?					
Rater 1&2	Body functions—Mental functions	Specific mental functions	b152 Emotional functions	b1522 Range of emotion	Mental functions that produce the spectrum of experience of arousal of affect or feelings such as love, hate, anxiousness, sorrow, joy, fear and anger
6. How often during the last 2 weeks have you felt worn out or low in energy?					
Rater 1&2	Body functions—Mental functions	Global Mental function	b130 Energy and drive functions	b1300 Energy level	General mental functions of physiological and psychological mechanisms that cause the individual to move towards satisfying specific needs and general goals in a persistent manner—Mental functions that produce vigour and stamina
7. How happy, satisfied, or pleased have you been with your personal life during the last 2 weeks?					
Rater 1&2	Body functions	Mental functions—Specific mental functions	b152 Emotional functions	b1520 Appropriateness of emotion	Mental functions that produce congruence of feeling or affect with the situation, such as happiness at receiving good news
8. In general, how often during the last 2 weeks have you felt restless, or as if you were having difficulty trying to calm down?					
Rater 1&2	Body functions	Mental functions Specific mental functions	b152 Emotional functions	b1521 Regulation of emotion	Mental functions that control the experience and display of affect
9. How much shortness of breath have you experienced during the last 2 weeks while doing your day-to-day physical activities?					
Rater 1&2	Body functions	Functions of the cardiovascular, haematological immunological and respiratory system	b460 Sensations associated with cardiovascular and respiratory functions		Sensations such as missing a heartbeat, palpitation and shortness of breath
10. How often during the last 2 weeks have you felt tearful or like crying?					
Rater 1&2	Body functions	Mental functions Specific mental functions	b152 Emotional functions	b1521 Regulation of emotion	Mental functions that control the experience and display of affect
11. How often during the last 2 weeks have you felt as if you are more dependent than you were before your heart problem?					
Rater 1&2	Not classified—NC				
12. How often during the last 2 weeks have you felt that you were unable to do your usual social activities or social activities with your family?					
Rater 1	Activities and participation	Community, social and civic life	d920—Recreation and leisure	d9205 Socializing	Engaging in informal or casual gatherings with others, such as visiting friends or relatives or meeting informally in public places
Rater 2	Activities and participation—Interpersonal interactions and relationships	General interpersonal interactions	d710 Basic interpersonal interactions d720 Complex interpersonal interactions		Usual social activities, social activities with your family
13. How often during the last 2 weeks have you felt as if others no longer have the same confidence in you as they did before your heart problem?					
Rater 1&2	Environmental factors	Attitudes	e410 Individual attitudes of immediate family members e415 Individual attitudes of extended family members e420 Individual attitudes of friends e425 Individual attitudes of acquaintances, peers, colleagues, neighbours and community members		General or specific opinions and beliefs of immediate family members about the person or about other matters (e.g. social, political and economic issues), that influence individual behaviour and actions)
14. How often during the last 2 weeks have you experienced chest pain while doing your day-to-day activities?					
Rater 1&2	Body functions	Sensory functions and pain—Pain	b280 Sensation of pain	b2801 Pain in body part	b28011 Pain in chest
15. How often during the last 2 weeks have you felt unsure of yourself or lacking in self-confidence?					
Rater 1&2	Body function—Mental function	Global mental function	b126 Temperament and personality functions	b1266 Confidence	Mental functions that produce a personal disposition that is self-assured, bold and assertive, as contrasted to being timid, insecure and self-effacing
16. How often during the last 2 weeks have you been bothered by aching or tired legs?					
Rater 1&2	Body functions	Sensory functions and pain—Pain	b280 Sensation of pain	b2801 Pain in body part	b28015 Pain in lower limb
17. During the last 2 weeks, how much have you been limited in doing sports or exercise as a result of your heart problem?					
Rater 1&2	Body functions	Functions of the cardiovascular, haematological immunological and respiratory system	b455 Exercise and tolerance functions	b4550 General physical endurance	
18. How often during the last 2 weeks have you felt apprehensive or frightened?					
Rater 1&2	Body functions—Mental functions	Specific mental functions	b152 Emotional functions	b1522 Range of emotion	Mental functions that produce the spectrum of experience of arousal of affect or feelings such as love, hate, anxiousness, sorrow, joy, fear and anger
19. How often during the last 2 weeks have you felt dizzy or lightheaded?					
Rater 1&2	Body functions	Functions of the cardiovascular, haematological immunological and respiratory system—Additional functions and sensations of the cardiovascular and respiratory systems	b469 Additional functions and sensations of the cardiovascular and respiratory systems, other specified and unspecified		
20. In general, during the last 2 weeks how much have you been restricted or limited as a result of your heart problem?					
Rater 1&2	Not definable—ND				
21. How often during the last 2 weeks have you felt unsure as to how much exercise or physical activity you should be doing?					
Rater 1&2	Body functions	Functions of the cardiovascular, haematological immunological and respiratory system	b455 Exercise and tolerance functions	b4550 General physical endurance	
22. How often during the last 2 weeks have you felt as if your family is being over-protective toward you?					
Rater 1&2	Environmental factors	Attitudes	e410 Individual attitudes of immediate family members		General or specific opinions and beliefs of immediate family members about the person or about other matters (e.g. social, political and economic issues), that influence individual behaviour and actions)
23. How often during the past 2 weeks have you felt as if you were a burden to others?					
Rater 1&2	Environmental factors	Attitudes	e410 Individual attitudes of immediate family members e415 Individual attitudes of extended family members e420 Individual attitudes of friends e425 Individual attitudes of acquaintances, peers, colleagues, neighbours and community members		General or specific opinions and beliefs…about the person or about other matters
24. How often during the past 2 weeks have you felt excluded from doing things with other people because of your heart problem?					
Rater 1&2	Activities and participation	Community, social and civic life	d910—Community life	d9109 Community life unspecified	
25. How often during the past 2 weeks have you felt unable to socialize because of your heart problem?					
Rater 1&2	Activities and participation	Community, social and civic life	d920—Recreation and leisure	d9205 Socializing	Engaging in informal or casual gatherings with others, such as visiting friends or relatives or meeting informally in public places
26. In general, during the last 2 weeks how much have you been physically restricted or limited as a result of your heart problem?					
Rater 1&2	Not definable—ND				
27. How often during the last 2 weeks have you felt your heart problem limited or interfered with sexual intercourse?					
Rater 1&2	Activities and participation	Interpersonal interactions and relationships—particular interpersonal relationships	d770 intimate relationships	d7702 sexual relationships	

### The 12-item short form health survey (SF-12)

For this measure, results from the linking guidelines paper are reported as the SF-12 was the illustrated example, reproduced in Table S4 [49]. All items of SF-12 were linked to the ICF category activities and participation.

All the SF-12 domains were mapped to ICHOM global sets for coronary artery disease, heart valve disease, heart failure and atrial fibrillation as described in see Table 3.

### MacNew health-related quality of life questionnaire

Items of the MacNew were linked to ICF categories except items 3 and 11, which were *not classified* and items 20 and 26, which were *not definable* (see Table 4). Thirteen items were linked to the Body Functions category, and chapters *mental functions* (1, 4, 5, 6, 7, 8, 10, 18), *functions of the cardiovascular, haematological, immunological, and respiratory system* (9, 19, 21), and s*ensory functions* (14, 16). Five items were linked to the category Activities and Participation, chapters *community, social and civic life* (12, 17, 24, 25) and *interpersonal interactions and relationships—particular interpersonal relationships* (item 27). Items 13 and 22, were linked to the Environmental Factors category chapter *attitudes*. The level of agreement between reviewers was 96% on the categories, 93% on the chapters and level 1 with 89% agreement on level 2.

All items of the MacNew were mapped to ICHOM global sets for coronary artery disease, heart valve disease, heart failure and atrial fibrillation as demonstrated in see Table 3.

### Euroqol 5-dimensions (EQ-5D)

All domains of the EQ-5D were linked to ICF categories (see Table 5). The mobility, self-care and usual activities domains were linked to the Activities and Participation category and chapters *mobility*, *self-care* and *general tasks and demands,* respectively. Pain/discomfort and anxiety/depression domains were linked to the Body Function category, chapters *sensory functions and pain* and *mental functions,* respectively. Agreement between reviewers was 90% for the categories, 80% for the chapters and 70% for level 1.

**Table 5:** Linking EQ-5D to the International Classification of Functioning, Disability and Health (ICF)

Rater	PROM item	ICF category	Level 1	Level 2	Level 3
Rater 1&2	Mobility I have no problems in walking about I have slight problems in walking about I have moderate problems in walking about I have severe problems in walking about I am unable to walk about	Activities and participation	Mobility – Walking and Moving	D450 – Walking	D4500-Walking short distances D4501-Walking long distances D4502-Walking on different surfaces D4503-Walking around obstacles D4508-Other walking unspecified D4509-Other specified walking
Rater 1&2	Self-care I have no problems washing or dressing myself I have slight problems… I have moderate problems. I have severe problems… I am unable to wash or dress myself	Activities and participation –	Self-care	D510 – washing oneself D540 – Dressing	D5400-Putting on clothes D5401-Taking off clothes D5402-Putting on footwear D5403-Taking off footwear D5404-Choosing appropriate clothing
Rater 1	Usual activities (e.g. work, study, housework, family or leisure activities)	Activities and participation –	General tasks and demands -	D230—Carrying out daily routine	D2301 – managing daily routine
	Work	Activities and participation	General tasks and demands—major life areas-work and employment	d850—remunerative employment	
	Study	Activities and participation	General tasks and demands – Education	D835—education life	
	Housework	Activities and participation	General tasks and demands—Domestic life	D640—doing housework	
	Leisure activities	Activities and participation	General tasks and demands – community social and civic life	d920—recreation and leisure	
	Family	Activities and participation—Interpersonal interactions and relationships	General interpersonal interactions	d710 Basic interpersonal interactions d720 Complex interpersonal interactions	
Rater 2		Activities and participation	General tasks and demands	d210 Undertaking a single task	d2100 Undertaking a simple task d2101 Undertaking a complex task d2102 Undertaking a single task independently d2103 Undertaking a single task in a group d2108 Undertaking single tasks, other specified d2109 Undertaking single tasks, unspecified
				d220 Undertaking multiple tasks	d2200 Carrying out multiple tasks d2201 Completing multiple tasks d2202 Undertaking multiple tasks independently d2203 Undertaking multiple tasks in a group d2208 Other specified undertaking multiple tasks d2209 Undertaking multiple tasks, unspecified
				d230 Carrying out daily routine	d2301 Managing daily routine d2302 Completing the daily routine d2303 Managing one's own activity level d2304 Adapting to changes in daily routine d2308 Other specified carrying out daily routine d2309 Carrying out daily routine, unspecified
				d240 Handling stress and other psychological demands	d2400 Handling responsibilities d2401 Handling stress d2402 Handling crisis d2408 Other specified handling stress and other psychological demands d2409 Handling stress and other psychological demands, unspecified
			Education	d835 Education life	
			Work and employment	d850 Remunerative employment	
				d298 Other specified general tasks and demands	
				d299 General tasks and demands, unspecified	
			Major life areas—Acquisition of necessities	d610 Acquiring a place to live d620 Acquisition of goods and services d629 Acquisition of necessities, other specified and unspecified	
			Domestic life		
Rater 1&2	Pain/Discomfort I have no pain or discomfort I have slight pain or discomfort I have moderate pain or discomfort I have severe pain or discomfort I have extreme pain or discomfort	Body functions	Sensory functions and pain—Pain	b280—sensation of pain	
Rater 1&2	Anxiety/Depression I am not anxious I am slightly anxious I am moderately anxious I am severely anxious I am extremely anxious	Body functions	Mental functions	b152 Emotional functions	b1528 Other specified emotional functions -*anxiety/depression* b1522 Range of emotion* Mental functions that produce the spectrum of experience of arousal of affect or feelings such as love, hate, anxiousness, sorrow, joy, fear and anger*

All domains of the EQ-5D were mapped to ICHOM global sets for coronary artery disease, heart valve disease, heart failure and atrial fibrillation (see Table 3).

## Discussion

### Main findings

Nine utility-based PROMs validated for application in populations undergoing cardiac rehabilitation and secondary prevention programs were identified; the German [42] and English [41] translations of SF-12, the German translation of EQ-5D-3L [43] and EQ-5D-5L [44], the Italian [38], Portuguese [37], French [36], Persian [35] and German [39, 40] translations of the MacNew heart disease HRQoL questionnaire.

The quality of evidence for responsiveness and hypothesis testing of the German version of the SF-12 [42] was moderate. The quality of evidence for structural validity, reliability, criterion validity, hypothesis testing, and responsiveness of the German version of EQ-5D-5L [44] was moderate. The quality of evidence for structural validity, internal consistency and hypothesis testing, reliability and responsiveness of the German version of MacNew [39, 40] was high. The quality of evidence for measurement properties of the following PROMs in a population undergoing cardiac rehabilitation and secondary prevention programs was low; English version of SF-12 [41], German translation of EQ-5D-3L [43], Portuguese [37], French [36], Italian [38] and Persian [35] translations of the MacNew heart disease questionnaire.

For all PROMs, linking was predominantly to the activities and participation category of the ICF. All the PROMs domains were matched onto similar constructs from the ICHOM global sets.

### Discussion of findings

Several studies have reviewed the literature to identify PROMs used in patients with cardiovascular disease [50, 51], however, this is the first study to specifically consider utility-based PROMs and their measurement properties in patients undergoing a cardiac rehabilitation and secondary prevention program. Since improvement in HRQoL is expected with CR and cost-effectiveness assessment of models and modes of delivery for CR, such as home-based and web-based CR, is key to inform implementation into practice, it is important to identify the best PROMs for assessing these outcomes. Like Thompson et al., 2016 [50], our review identified the disease-specific MacNew, which has been validated and can be applied across different cardiac populations and both the generic PROMs EQ-5D and SF-12 [50]. Our findings are particularly important to inform the choice of PROMs for application in cost-utility analysis studies, which are increasingly a preferred type of analysis recommended by decision-making bodies like NICE in the UK and PBAC in Australia [11, 12]. A recent review of the national health technology assessment (HTA) guidelines from these bodies revealed the prevalence of the generic utility-based PROMs as recommended for use in cost-utility analysis [52]. However, there is potential for additional validated PROMS to be applicable using mapping algorithms to calculate utility scores from responses to non-preference-based disease-specific PROMs. Mapping algorithms to the generic EQ-5D-5L have been developed for some PROMs like the MacNew heart disease HRQoL questionnaire [53, 54], the Kansas City Cardiomyopathy Questionnaire (KCCQ) [55], and the Minnesota Living with Heart Failure Questionnaire (MLHFQ) [56], and have been applied in cost-utility studies [55, 57].

Thompson et al., 2016 [50] highlighted the importance of measurement properties, specifically reliability, validity and responsiveness, when choosing a PROM to be used in cardiovascular disease. This review found moderate level evidence for responsiveness and validity of the German versions of the SF-12 [42], EQ-5D-5L [44] and MacNew heart disease questionnaire [39, 40] in a population undergoing a cardiac rehabilitation and secondary prevention program. These PROMs' reliability (test re-test reliability and internal consistency) is also reported. Responsiveness of the German version of SF-12 in a study assessing predictors of returning to work six months following cardiac rehabilitation and secondary prevention programs reported a moderate standardised effect size of 0.53 and 0.51 for the physical (PCS) and mental (MCS) component scales [58]. The majority (40%) of patients in this study had acute coronary syndrome (ACS), and 8% had undergone coronary artery bypass grafting (CABG). The standard response mean reported by Muller-Nordhorn et al., 2004 [42], identified in this review, for patients who had undergone CABG were PCS = 0.63 and MCS = 0.60 while for those undergoing CR following a myocardial infarction/ACS were MCS = − 0.18 and PCS = − 0.05. Due to the disproportionate distribution of CABG patients in that sample [58], the results of these two studies are not comparable for CABG and are dissimilar for myocardial infarction or ACS, highlighting the need for further studies on the responsiveness of this PROM in this population.

In their scoping review and mapping of heart disease-specific PROMs to the ICF, Alguren et al., 2020 [51] identified 34 PROMs whose items were linked to ICF categories of body function, activities and participation and environmental factors. Similarly, in our review, the heart disease specific MacNew was linked to body function (13 items) and activities and participation (5 items). All items of the EQ-5D were linked to similar ICF categories and chapters in this review, like Cieza and Stucki, 2005 [59]. Mobility was linked to b450 (walking); self-care was linked to d510 (washing oneself) and d540 (dressing); usual activities to d2301 (managing daily routine), d850 (remunerative employment), d835 (education life), d640 (doing housework) and d920 (recreation and leisure); pain/discomfort to b280 (sensation of pain) and anxiety/depression to b152 (emotional functions), b1528 (other specified emotional functions) and b1522 (range of emotion).

### Limitations

Although extensive searches were conducted, there were insufficient studies to undertake a meta-analysis of the measurement properties. Several language translations of the MacNew were identified, but only one study reported measurement properties of each version except the German version with two studies. This highlights the need for future studies to assess measurement properties of various translations of utility-based PROMs to guide recommendations for inclusion in health economic modelling studies assessing interventions in the different environments of delivery of cardiac rehabilitation and secondary prevention programs.

There are several limitations of the COSMIN guidelines noted in the literature regarding reporting of the assessment categories for the measurement properties [60]. This is classified as + *sufficient, ? Indeterminate—insufficient* and refers to the design and reporting of the validation studies but may be interpreted as the quality of the PROM, which is not the case [60]. Commentators recommend more clarity in the guidelines regarding this rating as it affects the confidence users will have in the given PROM. In addition, completing the risk of bias assessment and the GRADE assessment takes a significant amount of time and requires a more than basic understanding of psychometrics [61].

### Implications for practice

The EQ-5D-5L, SF-12 and MacNew heart disease questionnaire are linked to ICF categories and ICHOM global sets for CVD, demonstrating their suitability in a population experiencing any form of disability and cardiovascular disease.

This review has highlighted significant gaps in the literature on validation studies for utility-based PROMs in this population and the need for future research to validate these PROMs in patients undergoing cardiac rehabilitation and secondary prevention programs.

## Conclusion

This review has identified three PROMs that can generate health state utility values, validated for cardiac rehabilitation and secondary prevention programs: the German version of the generic EQ-5D and SF-12 and the heart disease-specific MacNew HRQoL questionnaire. The PROMs were predominantly linked to ICF categories of Body Function, and Activities and Participation, and matched to all ICHOM global sets. However, with only the German versions of these measures validated in cardiac rehabilitation and secondary prevention programs, it highlights the need for future larger studies to validate the different language translations of PROMs and provide options for use in this population.

## Supplementary Information

Below is the link to the electronic supplementary material.

Supplementary material 1 (DOCX 37 kb)

Supplementary material 2 (DOCX 24.0 kb)

Supplementary material 3 (DOCX 23.4 kb)

Supplementary material 4 (DOCX 31.5 kb)

Supplementary material 5 (DOCX 27 kb)
