# Supplementary material for: Measurement properties of utility-based health-related quality of life measures in cardiac rehabilitation and secondary prevention programs: a systematic review
Source: Qual Life Res. 2024 Jul 3;33(9):2299–320. doi: 10.1007/s11136-024-03657-5 (PMC11390805; doi:10.1007/s11136-024-03657-5)
Supplement: Supplementary file 1 — Supplementary material 1 (DOCX 37 kb) [file 11136_2024_3657_MOESM1_ESM.docx]

### Figure S1: PRISMA diagram for study screening and identification

**Identification**

References from other sources **(n =47)**

Grey literature (n =47)

Studies screened **(n = 3083)**

Full texts retrieval **(n = 39)**

Studies assessed for eligibility **(n = 36)**

Studies excluded **(n = 3044)**

Full texts not found **(n = 3)**

Studies excluded **(n = 26)**

No PROM used (n = 1)

Wrong outcomes (n = 1)

Not Cardiac rehab (n = 13)

Not utility-based PROM (n = 11)

Studies included in review

**(n = 10)**

**Screening**

Studies from databases/registers

**(n = 4722)**

References removed **(n=1686)**

Duplicates identified manually (n=1622)

Duplicates identified by Covidence (n=39)

Other reasons (n=25)
