# Supplementary material for: Measurement properties of utility-based health-related quality of life measures in cardiac rehabilitation and secondary prevention programs: a systematic review
Source: Qual Life Res. 2024 Jul 3;33(9):2299–320. doi: 10.1007/s11136-024-03657-5 (PMC11390805; doi:10.1007/s11136-024-03657-5)
Supplement: Supplementary file 2 — Supplementary material 2 (DOCX 24.0 kb) [file 11136_2024_3657_MOESM2_ESM.docx]

**Table S1: COSMIN definition of measurement properties and statistical tests to assess them**

| **Property** | **Definition [1]** | **Statistical tests [2]** |
| --- | --- | --- |
| Internal consistency | Degree on interrelatedness within the items of an instrument | Cronbach’s alpha  Kuder-Richardson Formula 20 (KR-20) |
| Reliability | Tests of reliability of an instrument consider the ability of an instrument to reproduce a result consistently in time and space | Same person over time = test-retest reliability  By different persons on the same occasion = inter-rater reliability  By the same persons on different occasions = intra-rater reliability |
| Measurement error | The systematic and random error of a patient’s score that is not attributed to true changes in the construct to be measured | Standard error of measurement (SEM), Smallest detectable change (SDC) and Limits of Agreement (LOA) |
| Content validity | assesses whether the items/questions within an instrument measure the concepts or domains they are reported to measure | No statistical test |
| Criterion validity | The degree to which the scores generated by the instrument reflect a given gold standard* | Pearson’s and Spearman’s correlation coefficients  **There is no gold standard for HR-PROMs or MAUIs, as such, the routinely assessed types of validity for these instruments are content validity and construct validity [3].* |
| Construct validity | The degree to which the scores are consistent with hypotheses based on the assumption that  the instrument validly measures the purported construct | No statistical test |
| Responsiveness | Responsiveness is the ability of an instrument to detect change over time in the construct its reported to measure | Correlations between change scores  The area under the receiver operator curve (ROC). |

*Definitions are adapted from the COSMIN taxonomy and definitions of measurement properties for health-related patient-reported outcomes [1].*

1. Mokkink, L. B., Terwee, C. B., Patrick, D. L., Alonso, J., Stratford, P. W., Knol, D. L., Bouter, L. M., & de Vet, H. C. (2010). The COSMIN study reached international consensus on taxonomy, terminology, and definitions of measurement properties for health-related patient-reported outcomes. Journal of clinical epidemiology, 63(7), 737-745.

2. Mokkink, L. B., Terwee, C. B., Patrick, D. L., Alonso, J., Stratford, P. W., Knol, D. L., Bouter, L. M., & de Vet, H. C. (2010). The COSMIN checklist for assessing the methodological quality of studies on measurement properties of health status measurement instruments: an international Delphi study. Qual Life Res, 19(4), 539-549.

3. Mokkink, L. B., Terwee, C. B., Knol, D. L., Stratford, P. W., Alonso, J., Patrick, D. L., Bouter, L. M., & de Vet, H. C. W. (2010). The COSMIN checklist for evaluating the methodological quality of studies on measurement properties: A clarification of its content. BMC Medical Research Methodology, 10(1), 22.
