# Supplementary material for: Measurement properties of utility-based health-related quality of life measures in cardiac rehabilitation and secondary prevention programs: a systematic review
Source: Qual Life Res. 2024 Jul 3;33(9):2299–320. doi: 10.1007/s11136-024-03657-5 (PMC11390805; doi:10.1007/s11136-024-03657-5)
Supplement: Supplementary file 3 — Supplementary material 3 (DOCX 23.4 kb) [file 11136_2024_3657_MOESM3_ESM.docx]

**Table S2: Medline search strategy – Medline via Ovid**

**Limits**: Humans, English language

|  | **Searches** | **Results** |
| --- | --- | --- |
| 1 | "Quality of Life"/ or quality-adjusted life years/ | 260686 |
| 2 | ("quality of life" or QoL or HRQOL or HRQL or HRQOL or QL or health related QOL or hql or hqol or h-qol or hr-qol or quality adjusted life or qaly* or qald* or qale* or qtime* or disability adjusted life or daly* or health utilit* or health outcomes or patient outcome or functioning or activit* or participation or health status or functional status).tw,kw. | 4136783 |
| 3 | (Quality adj2 (well-being or wellbeing)).tw,kw. | 2542 |
| 4 | (qwb or "quality of wellbeing" or "quality of well-being").tw,kw. | 537 |
| 5 | 1 or 2 or 3 or 4 | 4190847 |
| 6 | exp patient reported outcome measures/ | 12174 |
| 7 | ("preference-based instrument" or "multi-attribute utility" or MAU or "preference valu*" or "preference scale*" or Health Utilit* or HUI-2 or HUI2 or HUI Mark 2 or HUI-3 or HUI3 or HUI Mark 3 or "quality of wellbeing scale*" or QWB* or "assessment of quality of life" or AQoL* or EQ 5D* or EQ5D* or EQ-5D or SF 6D* or SF6D* or SF-6D or SF 36* or SF36* or SF-36 or 15D quality adjusted life or qaly* or qald* or qale* or qtime* or disability adjusted life or daly*).tw,kw. | 58596 |
| 8 | 6 or 7 | 69668 |
| 9 | (instrumentation or methods).sh. | 231754 |
| 10 | (validation study or comparative study).pt. | 2000279 |
| 11 | exp Psychometrics/ | 85619 |
| 12 | psychometr*.tw,kw. | 58937 |
| 13 | (clinimetr* or clinometr*).mp. | 1455 |
| 14 | exp Outcome Assessment, Health Care/ | 1298436 |
| 15 | exp Observer Variation/ | 44707 |
| 16 | exp Health Status Indicators/ | 338540 |
| 17 | exp Reproducibility of Results/ | 452369 |
| 18 | exp Discriminant Analysis/ | 11748 |
| 19 | (outcome assessment or observer variation or reproducib* or reliab* or unreliab* or valid* or coefficient of variation or coefficient or homogeneity or homogeneous or internal consistency or (cronbach* and (alpha or alphas)) or (item and (correlation* or selection* or reduction*))).tw,kw. | 1834802 |
| 20 | (outcome measure* or agreement or precision or imprecision or precise values).mp. | 758132 |
| 21 | ((reliab* and "test or retest") or stability or "test-retest" or "test and retest").tw,kw. | 545896 |
| 22 | (interrater or inter-rater or intrarater or intra-rater or intertester or inter-tester or intratester or intra-tester or interobserver or inter-observer or intraobserver or intra-observer or intertechnician or inter-technician or intratechnician or intra-technician or interexaminer or inter-examiner or intraexaminer or intra-examiner or interassay or inter-assay or intraassay or intra-assay or interindividual or inter-individual or intraindividual or intra-individual or interparticipant or inter-participant or intraparticipant or intra-participant or kappa or kappas).tw,kw. | 197609 |
| 23 | ((repeatab* or replicab* or repeated) and (measure or measures or findings or result or results or test or tests)).mp. | 259133 |
| 24 | (generaliza* or generalisa* or concordance or (intraclass and correlation*) or Discriminative or known group or factor analysis or factor analyses or factor structure or factor structures or dimension* or subscale* or ((multitrait or scaling) and (analysis or analyses)) or item discriminant or interscale correlation* or error or errors or individual variability or interval variability or rate variability or (variability and (analysis or values)) or (uncertainty and (measurement or measuring)) or "standard error of measurement" or sensitiv* or responsive* or interpretab* or (limit and detection) or minimal detectable concentration).tw,kw. | 3113582 |
| 25 | ((minimal or minimally or clinical or clinically) and (important or significant or detectable or change or difference)).tw,kw. | 1606568 |
| 26 | ((small* and (real or detectable) and (change or difference)) or meaningful change or ceiling effect or floor effect or item response model or IRT or rasch or DIF or differential item functioning or computer adaptive testing or item bank or cross-cultural equivalence).tw,kw. | 25431 |
| 27 | or/9-26 | 9284706 |
| 28 | cardiovascular diseases/ or exp cardiovascular abnormalities/ or heart diseases/ or exp arrhythmias, cardiac/ or exp cardiac conduction system disease/ or exp cardiomyopathies/ or heart aneurysm/ or exp heart arrest/ or exp heart failure/ or exp heart rupture/ or exp heart valve diseases/ or exp myocardial ischemia/ or exp ventricular dysfunction/ or exp ventricular outflow obstruction/ or exp aneurysm/ or exp arterial occlusive diseases/ or exp arteriovenous malformations/ or exp peripheral vascular diseases/ or exp Cardiovascular Surgical Procedures/ or exp Hyperlipid?emia, Familial combined/ or exp assisted circulation/ or exp pacemaker, artificial/ | 1843093 |
| 29 | ((Heart or cardiac or coronary or cardiovascular) adj2 (disease* or conduction disease* or attack or aneurysm or arrest or death or failure or surgery or surgical or surgeries or transplant* or valve disease or bypass or graft or procedure or rhythm problems or arrhythmias)).tw,kw. | 833419 |
| 30 | (Myocardial adj5 (isch?emia or infarct* or ST elevation or non-ST elevation)).kw,kf. | 32789 |
| 31 | (aneurysm or atrial fibrillation or ventricular dysfunction or ventricular outflow obstruction or arterial occlusive disease* or arteriovenous malformation* or peripheral vascular disease* or Implantable cardioverter defibrillator or ICD or angina or percutaneous coronary intervention or PCI or CABG or Post-revasculari?ation procedure* or STEMI or AMI or non-STEMI or stent or angioplasty or PTCA or familial hypercholesterol?emia or ventricular assist device or assisted circulation or permanent pacemaker).tw,kw. | 514717 |
| 32 | 28 or 29 or 30 or 31 | 2229676 |
| 33 | rehabilitation/ or cardiac rehabilitation/ or exp exercise therapy/ or occupational therapy/ or rehabilitation, vocational/ or telerehabilitation/ or consumer health information/ or health literacy/ or exp patient education as topic/ or psychology/ or psychology, positive/ or exp psychology, medical/ or psychology, social/ or psychotherapy/ or exp cognitive behavio?ral therapy/ or feedback, psychological/ or exp hypnosis/ or exp psychoanalytic therapy/ or psychosocial intervention/ or exp psychotherapeutic processes/ | 349893 |
| 34 | ((exercise or diet or lifestyle or occupational or educat* or rehab* or council* or psycholog* or psychosocial or psychotherapy*) adj4 (program* or intervention* or modification or therapy or process*)).tw,kf. | 255126 |
| 35 | (health literacy or consumer health information or telerehabilitation or patient education or CBT or cognitive behavio?r or pyscholog* feedback or hypnosis or psychoanalytic therapy).tw,kw. | 63108 |
| 36 | 33 or 34 or 35 | 575538 |
| 37 | 32 and 36 | 41054 |
| 38 | (cardiac adj2 rehab*).tw,kf. | 8154 |
| 39 | 37 or 38 | 44024 |
| 40 | 5 and 8 and 27 and 39 | 471 |
| 41 | (address or biography or case reports or comment or directory or editorial or festschrift or interview or lecture or legal case or legislation or letter or news or newspaper article or patient education handout or popular work or congress or consensus development conference or consensus development conference, NIH or practice guideline).pt. | 4689629 |
| 42 | 40 not 41 | 469 |
| 43 | limit 42 to English language | 441 |
| 44 | limit 43 to humans | 403 |
