# Supplementary material for: Measurement properties of utility-based health-related quality of life measures in cardiac rehabilitation and secondary prevention programs: a systematic review
Source: Qual Life Res. 2024 Jul 3;33(9):2299–320. doi: 10.1007/s11136-024-03657-5 (PMC11390805; doi:10.1007/s11136-024-03657-5)
Supplement: Supplementary file 4 — Supplementary material 4 (DOCX 31.5 kb) [file 11136_2024_3657_MOESM4_ESM.docx]

### Table S3: Characteristics of included studies

| **Study details** | | **Population** | | | **Instrument administration** | | | | | | **PROM compared to** |
| --- | --- | --- | --- | --- | --- | --- | --- | --- | --- | --- | --- |
| **PROM** | **Study design** | **Mean age (SD)** | **Number (%females)** | **Population description** | **Setting** | **Country** | **Language** | **Original translation** | **Administration** | **Measurement properties assessed** |  |
| SF-12[[36](#_ENREF_36)] | Cohort study | 63 years, SD=13 | N=65, 25% female | Hospital inpatients over the age of 18 years | Community/at home | Australia | English | English | Self-administered | Responsiveness | None |
| SF-12 [[35](#_ENREF_35)] | Cohort study | 60 years, SD=10 | n=2441, 22% female | CR after myocardial infarction, coronary artery bypass grafting, and percutaneous transluminal coronary angioplasty | Community/at home | Germany | German | English | Self-administered | Hypothesis testing, Responsiveness | SF-36 |
| EQ-5D-3L [[37](#_ENREF_37)] | Cross-sectional study | 55 years, SD=7.6 | n=114, 15% female | Patients with ACS (51% myocardial infarction, 42% coronary artery bypass grafting, 7% angina) | Hospital | Germany | German | English | Self-administered | Reliability, Hypothesis testing, Responsiveness | SF-36 & MacNew |
| EQ-5D-5L [[34](#_ENREF_34)] | Cohort study | 73.9 years, SD=10.2 | N=3225, 45.1% female | Primary care heart failure patient | Community/at home | Germany | German | English | Self-administered | Structural validity, Internal consistency, Hypothesis testing, | PHQ-9, HADS-A and HADS-D, PROMIS |
| MacNew [[41](#_ENREF_41)] | Non-randomised experimental study | 58.8 years, SD=11.6 | n=200, 15.3% female | Patients with an ACS diagnosis 2 months after their initial treatment. | Hospital | Portugal | Portuguese | English | Self-administered | Structural validity, Internal consistency, Reliability, Hypothesis testing, Responsiveness | SF-36, HADS |
| MacNew [[42](#_ENREF_42)] | Non-randomised experimental study | 58.6 years, SD=11.3 | n=323, 17.3% female |  | Community/at home | France | French | English | Self-administered | Structural validity, Internal consistency, Reliability, Hypothesis testing | SF-36, HADS |
| MacNew [[38](#_ENREF_38)] | Cross-sectional study | 64.8 years, SD=10.6 | n=298, 23.2% female | Patients with angina, myocardial infarction, or ischemic heart failure | Community/at home | Italy | Italian | English | Self-administered | Structural validity, Internal consistency, Hypothesis testing | SF-36, HADS |
| MacNew [[43](#_ENREF_43)] | Randomised controlled trial | 52.8 years, SD=9.3 | n=60, 33.3% female | Patients with HF referred to CR | Community/at home | Iran | Persian | English | Self-administered | Internal consistency, Reliability, Hypothesis testing, Responsiveness | SF-36 |
| MacNew [[40](#_ENREF_40)] | Non-randomised experimental study | 61.7 years, SD=11.5 | n=89, 15.7% female | German-speaking patients in two Austrian (N=66) and one Swiss (N=23) | Hospital | Austria and Switzerland | German | English | Self-administered | Structural validity, Internal consistency, Hypothesis testing, Responsiveness | SF-36 |
| MacNew [[39](#_ENREF_39)] | Cross-sectional study | Start of CR 69.8 years, SD=7.5 Six-month follow-up 69.8 years, SD=7.1 End of CR 69.7 years, SD=7.4 | Start of CR n=5692, 35.9% female Six-month follow-up n=3663, 34.2% female End of CR n=5169, 35.6% female | Patients in CR | Community/at home | Germany | German | English | Self-administered | Structural validity, Internal consistency, Hypothesis testing, Responsiveness | IRES-3 |

*PHQ-9=Patient Health Questionnaire depression subscale; HADS=Hospital Anxiety and Depression Scales and PROMIS=Patient Reported Outcomes Measurement Information System depression and anxiety items; SF-36=Short Form-36*
