# Supplementary material for: Measurement properties of utility-based health-related quality of life measures in cardiac rehabilitation and secondary prevention programs: a systematic review
Source: Qual Life Res. 2024 Jul 3;33(9):2299–320. doi: 10.1007/s11136-024-03657-5 (PMC11390805; doi:10.1007/s11136-024-03657-5)
Supplement: Supplementary file 5 — Supplementary material 5 (DOCX 27 kb) [file 11136_2024_3657_MOESM5_ESM.docx]

**Table S4: Linking SF-12 questionnaire to the International Classification of Functioning, Disability and Health (ICF)**

| **Item** | **ICF category** | **Additional information** |
| --- | --- | --- |
| 1. In general, would you say your health is: (Excellent Very Good Good Fair Poor) | nd-gh |  |
| 2. Does YOUR HEALTH NOW LIMIT YOU in these activities? If so, how much? | nd-gh |  |
| a. MODERATE ACTIVITIES, such as moving a table, pushing a vacuum cleaner, bowling, or playing golf: | Activities and Participation  (d430) Lifting and carrying objects  (d4451) Pushing  (d9201) Sports  (d9201) Sports | Moderate  A table  A vacuum cleaner Bowling  Playing golf |
| b. Climbing SEVERAL flights of stairs: | d4551 Climbing | Several ﬂights of stairs |
| 3. During the PAST 4 WEEKS have you had any of the following problems with your work or other regular activities AS A RESULT OF YOUR PHYSICAL HEALTH?  a. ACCOMPLISHED LESS than you would like:  b. Were limited in the KIND of work or other activities: | d850 Remunerative employment d230 Carrying out daily routine nd-ph |  |
| 4. During the PAST 4 WEEKS, were you limited in the kind of work you do or other regular activities AS A RESULT OF ANY EMOTIONAL PROBLEMS (such as feeling depressed or anxious)?  a. ACCOMPLISHED LESS than you would like  b. Didn’t do work or other activities as CAREFULLY as usual | d850 Remunerative employment  d230 Carrying out daily routine  b152 Emotional functions  (b152) Emotional functions  (b152) Emotional functions | Depressed  Anxious |
| 5. During the PAST 4 WEEKS, how much did PAIN interfere with your normal work (including both work outside the home and housework)? | b280 Sensation of pain  d850 Remunerative employment  d640 Doing housework |  |
| 6. The next three questions are about how you feel and how things have been DURING THE PAST 4 WEEKS. For each question, please give the one answer that comes closest to the way you have been feeling.  How much of the time during the PAST 4 WEEKS –  a. Have you felt calm and peaceful?  b. Did you have a lot of energy?  c. Have you felt downhearted and blue? | b152 Emotional functions  b1300 Energy level  b152 Emotional functions | Calm and peaceful  Downhearted and blue |
| 7. During the PAST 4 WEEKS, how much of the time has your PHYSICAL HEALTH OR EMOTIONAL PROBLEMS interfered with your social activities (like visiting with friends, relatives, etc.)? | nd-ph  b152 Emotional functions  d9205 Socializing |  |

nd-gh=not defined-general health; nd (not definable); nd-qol (not definable-quality of life); nd-ph=not definable-physical health
